# Perfil de Biomarcadores en Líquido Cefalorraquídeo en Casos de Enfermedad de Alzheimer con Presentación Atípica

**DOI:** 10.31083/RN36399

**Published:** 2025-05-22

**Authors:** Elisa Martínez Campos, Paula Tellechea Aramburo, Javier Sánchez Ruiz de Gordoa, Rosa Larumbe Ilundain

**Affiliations:** ^1^Servicio de Neurología, Hospital Universitario de Navarra, 31008 Pamplona, España

**Keywords:** demencia tipo Alzheimer, líquido cefalorraquídeo, proteína tau, Enfermedad de Alzheimer de inicio focal, afasia primaria progresiva, Alzheimer-type dementia (ATD), cerebrospinal fluid, tau proteins, Alzheimer’s disease, early onset, aphasia, primary progressive

## Abstract

**Introducción:**

Los biomarcadores de la Enfermedad de Alzheimer (EA) en líquido cefalorraquídeo (LCR) tienen un papel significativo en el diagnóstico precoz de fenotipos no-amnésicos. Se han descrito mayores niveles de tau total (t-tau) y tau fosforilada (p-tau) en casos atípicos de EA, aunque la existencia de un patrón diferencial sigue siendo controvertida. Nuestro objetivo fue estudiar las diferencias en el perfil de biomarcadores de EA en LCR en función del fenotipo.

**Material y Métodos:**

Revisión retrospectiva de características demográficas, tiempo hasta el diagnóstico, fenotipo clínico y biomarcadores “core” (péptido beta-amiloide 1-42 (Aβ1-42), t-tau, p-tau) de EA en LCR de pacientes valorados en nuestro centro entre 2019–2022.

**Resultados:**

57 fueron pacientes analizados (54% mujeres, edad media 67 años). 41 cumplían criterios diagnósticos de EA. De ellos, 10 (25%) presentaron un perfil atípico (50% afásico, 30% frontal, 20% mixto no amnésico). El grupo atípico presentó niveles mayores de t-tau (562,9 pg/mL vs 320,3 pg/mL, *p* = 0,021) y p-tau (81,5 pg/mL vs 37,7 pg/mL, *p* = 0,016) respecto al fenotipo amnésico, independientemente de la edad, sexo y tiempo hasta el diagnóstico.

**Conclusiones:**

En nuestro estudio, los casos atípicos presentaron valores de tau más elevados. Dichos resultados apoyan que estos fenotipos presentan daño cortical más precoz y grave que el fenotipo amnésico y subrayan la importancia de los biomarcadores en LCR como herramienta para la estratificación, predicción del curso clínico y personalización terapéutica de nuestros pacientes.

## Abstract (en)

**Background:**

Cerebrospinal fluid (CSF) biomarkers for Alzheimer’s disease (AD) are essential for the early identification of non-amnestic phenotypes. Increased levels of total tau (t-tau) and phosphorylated tau (p-tau) have been reported in atypical AD cases, although the specific pattern remains a subject of debate. This study aimed to evaluate CSF biomarker profiles in relation to clinical phenotype.

**Materials and Methods:**

A retrospective review was performed, analyzing demographic data, time to diagnosis, clinical phenotype, and core AD biomarkers (beta-amyloid peptide 1-42 (Aβ1-42), t-tau, p-tau) in CSF from patients evaluated at University Hospital in Navarra between 2019 and 2022.

**Results:**

The study included 57 patients (54% female, mean age 67 years), of whom 41 met AD diagnostic criteria. Among these, 10 patients (25%) presented with atypical phenotypes (50% aphasic, 30% frontal, 20% mixed non-amnestic). Compared with the amnestic phenotype, the atypical group exhibited significantly higher t-tau (562.9 pg/mL vs 320.3 pg/mL, *p* = 0.021) and p-tau (81.5 pg/mL vs 37.7 pg/mL, *p* = 0.016) levels, independent of age, sex, and time to diagnosis.

**Conclusions:**

Atypical cases demonstrated increased tau levels, suggesting earlier and more extensive cortical damage than the amnestic phenotype. These findings underscore the significance of CSF biomarkers in phenotypic differentiation, disease course prediction, and individualized treatment strategies for AD.

## 1. Introducción y Objetivos

La enfermedad de Alzheimer (EA) es una enfermedad neurodegenerativa progresiva, histopatológicamente caracterizada por el depósito de placas de amiloide y proteína tau en el cerebro [1]. El fenotipo clínico clásico corresponde al síndrome amnésico-afaso-apráxico, en el que predomina una afectación de la memoria como síntoma inicial. Existen fenotipos atípicos de la EA que se presentan con la afectación de otras funciones cognitivas en fases iniciales, con relativa preservación de la memoria. Entre ellos, se describe el fenotipo “afásico”, también conocido como “logopénico”, el fenotipo “posterior”, el fenotipo “disejecutivo” o el “conductual”, clásicamente agrupadas bajo el término “variante frontal” [2]. En los estudios histopatológicos de casos de EA con presentación atípica se ha descrito un depósito de tau neocortical mayor que en la forma clásica [3], siendo de predominio occipital en la variante posterior, o temporoparietal en la variante afásica [4].

El diagnóstico definitivo de EA es neuropatológico, aunque en los últimos años se han desarrollado biomarcadores (BM) que aumentan la certeza diagnóstica en vida. Según el esquema propuesto por Jack *et al*. [5], se establece una categorización ATN en el estudio de los biomarcadores de EA, donde “A” hace referencia al proceso de amiloidogénesis, “T” se relaciona con la taupatía y “N” es indicativa de la neurodegeneración. Existen diferentes tipos de biomarcadores: (1) los biomarcadores de imagen, como la resonancia magnética craneal (RM), la tomografía por emisión de positrones (PET) de Amiloide, la PET-tau y la PET con 18F-fluorodeoxiglucosa (PET-FDG); (2) los biomarcadores de líquido cefalorraquídeo (LCR), como la proteína beta-amiloide 42, la proteína tau total (t-tau) y tau fosforilada (p-tau) [2]; (3) los biomarcadores en sangre, como la proteína p-tau 217 o los neurofilamentos [6].

Se ha descrito la correlación entre los distintos biomarcadores de imagen y los fenotipos clínicos y neuropatológicos de EA [2]. No se han identificado diferencias en la distribución de placas en el PET-Amiloide entre los diferentes fenotipos. Sin embargo, se han observado patrones diferenciales en la PET-tau identificándose una mayor captación de radiotrazador neocortical en las variantes atípicas con respecto a la forma clásica. Se ha visto que esta hipercapatación metabólica se correlaciona con la sintomatología característica de cada variante. Los pacientes que debutan con alteración visuoespacial presentan una mayor intensidad de radiotrazador en el córtex occipital, mientras que en aquellos que debutan con alteraciones del lenguaje la intensidad es mayor a nivel de córtex parietotemporal izquierdo [7].

La mayoría de estudios realizados hasta la fecha no han encontrado diferencias significativas en el valor de proteína ß-amiloide en LCR del fenotipo amnésico vs los fenotipos atípicos de EA [2, 8, 9, 10, 11, 12]. Sin embargo, la relación entre el perfil de t-tau y p-tau en LCR y los diferentes fenotipos de EA no está claramente definida. Algunos autores han descrito la presencia de mayor concentración de t-tau en las variantes no amnésicas, observándose una mayor concentración de t-tau y p-tau en las variantes frontal y afásica con respecto al resto [8], y siendo la variante posterior la que presentaba concentraciones más bajas de todas [10]. Estos patrones no se han podido reproducir posteriormente [9].

Por este motivo, el objetivo de nuestro trabajo fue profundizar en las posibles diferencias en los valores de p-tau y t-tau en LCR entre los distintos fenotipos de EA.

## 2. Material y Métodos

Se realizó un estudio descriptivo retrospectivo de pacientes a los que les solicitaron biomarcadores en LCR en el Hospital Universitario de Navarra entre los años 2019–2022, previa aprobación del Comité de Ética (código de aceptación: PI_2023/71). Los motivos de la solicitud de extracción de BM en LCR fueron: edad temprana de inicio del cuadro clínico (<60 años), dudas diagnósticas, clínica atípica y/o posibilidad de participar en ensayos clínicos. Los criterios de exclusión fueron: imposibilidad de realizar punción lumbar, negatividad para péptido beta-amiloide 42 (Aβ42) o los cocientes tau total/Aβ42 positivo o tau fosforilada/Aβ42.

Las variables estudiadas fueron: edad, sexo, presencia de antecedentes familiares de EA, nivel educacional (estudios primarios/secundarios/superiores), tiempo desde el síntoma inicial hasta la determinación de BM en LCR, resultados de test neuropsicológicos (evaluación por neuropsicólogo entrenado/*Mini-Mental State Examination* (MMSE)), perfil de deterioro cognitivo (amnésico, multidominio con predominio amnésico, atípico (afásico/disejecutivo/posterior/multidominio no amnésico)) y perfil de biomarcadores de EA en LCR siguiendo el esquema A/T/(N), considerando como positivos aquellos con Aβ42 positivo o los cocientes tau total/Aβ42 positivo o tau fosforilada/Aβ42 positivo. El perfil del deterioro cognitivo se estableció en base a la historia clínica y los resultados de los test neuropsicológicos realizados al inicio del seguimiento.

Se utilizó el test de la χ^2^ para realizar comparaciones entre las variables categóricas (antecedentes familiares EA, grado de estudios), y el test no paramétrico de la U de Mann Whitney para el cálculo de diferencia de medias entre dos grupos (edad al diagnóstico, tiempo hasta primera consulta, tiempo hasta punción lumbar (PL)). Se realizó un modelo de regresión lineal múltiple, donde el perfil de deterioro (típico vs atípico) se utilizó como variable independiente y los valores de t-tau y p-tau como variables dependientes, ajustando por edad, sexo y tiempo hasta punción lumbar. El nivel de significación estadística fue *p* = 0,05. El programa estadístico utilizado fue IBM SPSS Statistics for Windows, Version 25,0 (IBM Corp., Armonk, NY, USA).

## 3. Resultados

**Fig. 1.**
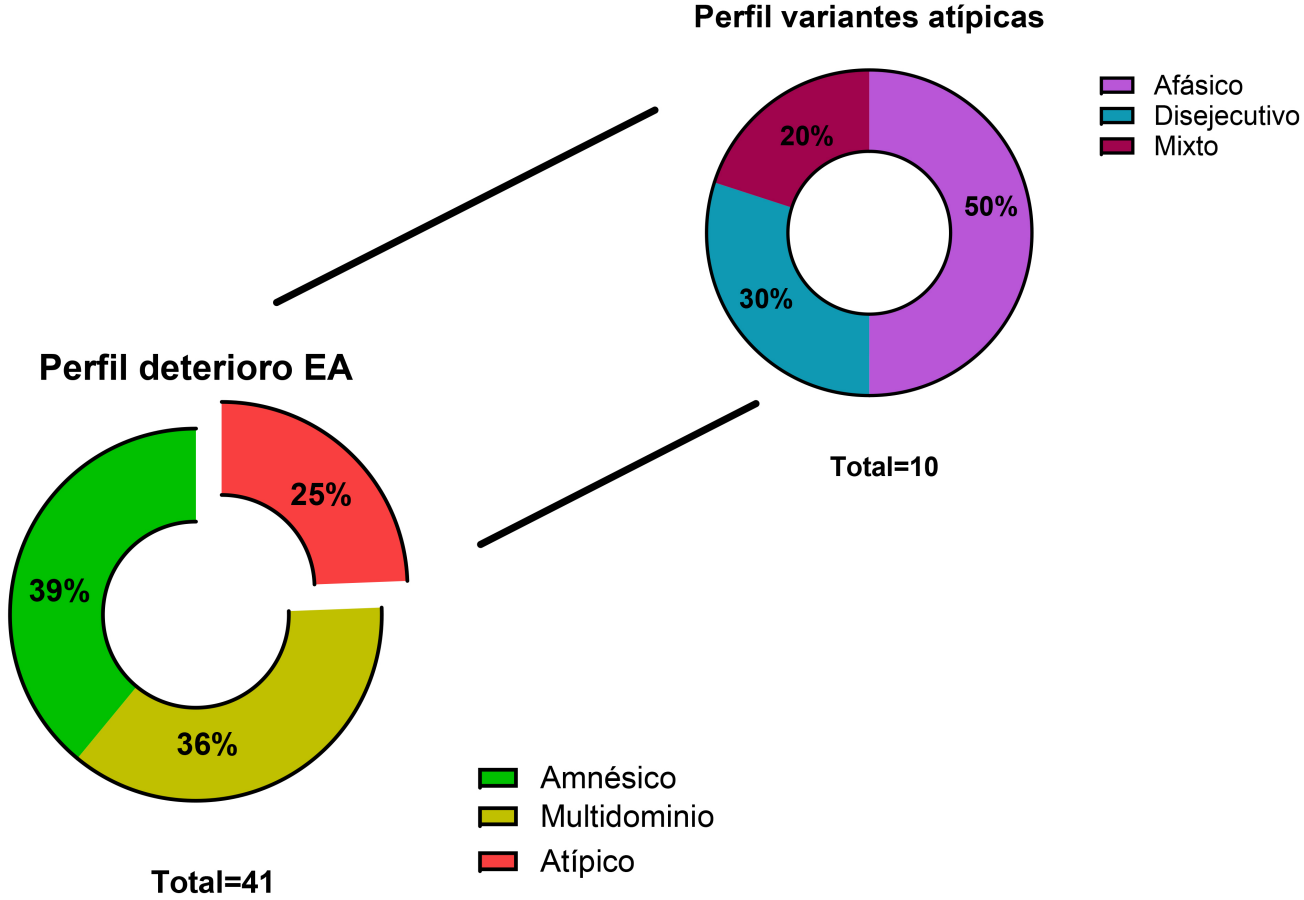
**Porcentaje de pacientes con EA confirmada por biomarcadores clasificados en los diferentes fenotipos en función de la clínica inicial y estudio neuropsicológico**. EA, enfermedad de Alzheimer.

Se realizó una PL para determinación de biomarcadores a 57 pacientes, siendo positivos para el perfil de EA 41 de ellos. 16 (39%) presentaron un perfil amnésico, 15 (36%) un deterioro multidominio con componente amnésico, y 10 (25%) un perfil atípico. Dentro de este último grupo, 5 (50%) se clasificaron como variante afásica, 3 (30%) como disejecutiva y 2 (20%) como afectación multidominio no amnésica en base a la historia clínica y los test neuropsicológicos (Fig. 1). En la Tabla 1 se resumen las variables demográficas analizadas y sus diferencias entre estos fenotipos, agrupados en dos perfiles: típico (amnésico y multidominio con componente amnésico) y atípico. El test MMSE solo se realizó en 18 pacientes de la muestra. El resto fueron evaluados por un neuropsicólogo entrenado, con realización de distintas baterías dirigidas a cada paciente a criterio del profesional (no adjuntado). El análisis de regresión lineal múltiple reveló niveles mayores de t-tau (562,9 pg/mL vs 320,3 pg/mL, *p* = 0,021) y p-tau (81,5 pg/mL vs 37,7 pg/mL, *p* = 0,016) en el grupo con fenotipo atípico con respecto al fenotipo amnésico, ajustando por sexo, edad y tiempo hasta PL (Tabla 2a,2b). La distribución de los patrones de BM de LCR según el esquema ATN en función de las variantes de EA en nuestra muestra se resume en la Tabla 3. No se identificaron diferencias significativas en los valores de t-tau y p-tau entre las diferentes variantes clínicas.

**Tabla 1.** **Características demográficas y epidemiológicas de los pacientes con EA en función de su fenotipo clínico**.

Característica		Perfil amnésico	Perfil atípico	Valor *p*
		(n = 31)	(n = 10)	
Edad media al diagnóstico (años)		70	68	0,68
Antecedentes familiares de EA (%)		13/31 (42%)	3/10 (30%)	0,50
Estudios	Primarios	16/31 (52%)	3/10 (30%)	0,25
	Secundarios	4/31 (13%)	3/10 (30%)	
	Superiores	9/31 (30%)	1/10 (10%)	
Tiempo hasta primera consulta (meses)		21	27	0,39
Tiempo hasta PL (meses)		14	11	0,69

PL, punción lumbar.

**Tabla 2a.** **Resultado de la regresión lineal múltiple para predecir el valor de las proteínas tau total y tau fosforilada (pg/mL) en LCR**.

Variable	Coeficiente (ß)	Error estándar	Valor t	Valor *p*
Constante	105,90	582,10	0,18	0,85
Sexo	25,26	85,06	0,29	0,76
Edad	2,83	8,24	0,34	0,73
EA atípica	246,50	98,69	2,49	0,021
Tiempo hasta PL (meses)	0,26	2,99	0,08	0,93

Resultados para la variante dependiente “tau total”; R^2^ = 0,14; R^2^ ajustada = 0,05; Error estándar de la estimación = 269,3; F (4, 36) = 1,583, *p* = 0,19; n = 41. LCR, líquido cefalorraquídeo.

**Tabla 2b.** **Resultado de la regresión lineal múltiple para predecir el valor de las proteínas tau total y tau fosforilada (pg/mL) en LCR**.

Variable	Coeficiente (ß)	Error estándar	Valor t	Valor *p*
Constante	18,36	95,58	0,19	0,84
Sexo	–6,91	13,97	0,49	0,62
Edad	0,24	1,35	0,18	0,85
EA atípica	45,05	16,21	2,78	0,016
Tiempo hasta PL (meses)	0,39	0,49	0,80	0,42

Resultados para la variante dependiente “tau fosforilada”; R^2^ = 0,18; R^2^ ajustada = 0,09; Error estándar de la estimación = 44,22; F (4, 36) = 2,09, *p* = 0,10; n = 41.

**Tabla 3.** **Distribución de los patrones de BM de LCR según el esquema ATN en función de las variantes de EA en nuestra muestra**.

	A+T-N-	A+T+N-	A+T+N+
Variante amnésica	36%	3%	61%
Variante disejecutiva	33%	33%	33%
Variante logopénica	40%	0%	60%
Variante multidominio no amnésico	100%	0%	0%

BM, biomarcadores; ATN, “A+” positividad para péptido beta-amiloide 42 (Aβ42), “T+” positividad para t-tau, “N+” positividad para p-tau.

## 4. Discusión

En nuestro estudio se observó una mayor cantidad de proteína t-tau y p-tau en pacientes con fenotipo atípico vs pacientes con fenotipo amnésico de EA. Estos resultados son consistentes con las publicaciones previas que sugieren un perfil distintivo de estos BM entre las diferentes variantes de EA [9, 12]. Pillai y colaboradores estimaron un riesgo del 7% de desarrollar un fenotipo no amnésico por cada aumento de 50 pg/mL de proteína t-tau en LCR [12]. Sin embargo, hasta la fecha no se han estandarizado puntos de corte fiables en los valores de t-tau y p-tau en LCR que permitan diferenciar las distintas variantes entre sí. Comparando entre variantes atípicas, Paterson *et al*. [8] identificaron la variante disejecutiva como aquella con mayor carga de tau total y fosforilada, seguida por la variante logopénica. Estas dos presentaciones son precisamente las más frecuentes entre los pacientes con clínica atípica en nuestro estudio. En contraste, otro estudio señaló que la variante posterior se asocia con niveles más bajos de tau, incluso inferiores a los observados en fenotipos amnésicos [10]. Respecto a Aβ42, no se han reportado diferencias significativas en el valor de esta proteína en LCR entre la variante amnésica y las variantes atípicas de EA, exceptuando el estudio de Paterson* et al*. [8] donde se observó un menor nivel de Aβ42 en LCR en el fenotipo frontal, comparado con la variante logopénica y la posterior. Sin embargo, consideramos que estas apreciaciones exceden el objetivo de nuestro trabajo, dada la poca representación de este fenotipo en nuestra muestra.

Estudios previos han asociado una mayor carga de proteína tau cortical con un comienzo más temprano de la enfermedad de Alzheimer y un deterioro cognitivo más pronunciado en las etapas iniciales [13], características frecuentemente observadas en las variantes atípicas [2, 11, 14]. Sin embargo, no hemos encontrado diferencias significativas en las variables sociodemográficas ni en los resultados de cognición entre los dos grupos de pacientes de nuestra muestra. Esto puede deberse a que la edad menor de 60 años se consideró criterio de inclusión a la hora de solicitar BM en LCR en nuestro estudio, lo que conlleva un sesgo de selección, habiéndose incluido pacientes más jóvenes con estadios clínicos más iniciales en el grupo de fenotipo amnésico. A pesar de esto, nuestros hallazgos sugieren la existencia de un correlato bioquímico diferencial en el LCR entre variantes clásicas y atípicas desde fases clínicas iniciales, evidenciado por niveles elevados de proteína tau en los fenotipos atípicos.

Este estudio tiene varias limitaciones; el reducido número de participantes disminuye la potencia estadística, aunque se mantuvieron diferencias significativas en los niveles de tau entre las variantes estudiadas. Por otro lado, la ausencia de casos de la variante posterior en nuestra muestra podría exagerar las diferencias entre las formas amnésica y atípica, considerando que algunos estudios han reportado niveles más bajos de tau en la variante posterior.

Por último, nuestro estudio no valora la progresión de los pacientes a largo plazo. Otros trabajos realizados previamente han evaluado la evolución longitudinal de los pacientes con diferentes fenotipos de EA y su correlato con BM de imagen, objetivándose una tasa de atrofia y depósito de tau cortical más precoces en las variantes atípicas, con una asociación negativa respecto a la edad, ya que los pacientes más jóvenes presentaron una progresión más rápida y agresiva [15, 16, 17]. Sin embargo, no hemos encontrado estudios que hagan lo mismo con BM en LCR. Se requieren investigaciones adicionales con períodos de seguimiento más extensos para determinar si los niveles elevados de tau en LCR se correlacionan con un peor pronóstico durante el curso de la enfermedad.

## 5. Conclusiones

En nuestro estudio se encontró un perfil de biomarcadores distintivo entre los fenotipos atípicos de EA y la presentación clásica, con niveles significativamente más elevados de tau total y tau fosforilada en el LCR de pacientes con variante frontal y variante logopénica. Este patrón diferencial es congruente con los hallazgos de neuroimagen y las características histopatológicas de neurodegeneración asociadas a estas variantes. La presencia de este perfil bioquímico particular en el LCR podría ser indicativa de un proceso de neurodegeneración cortical más agresivo y temprano en comparación con el fenotipo amnésico clásico. Estos hallazgos subrayan la importancia de los biomarcadores en LCR como herramientas potenciales para la estratificación de pacientes, la predicción del curso clínico y, posiblemente, la personalización de estrategias terapéuticas en el futuro. Se requieren estudios longitudinales adicionales para validar el valor predictivo de estos biomarcadores y su aplicabilidad en la práctica clínica.

## Data Availability

Los conjuntos de datos utilizados y/o analizados durante el presente estudio están disponibles a pedido razonable del autor correspondiente.

## Divulgación

Este trabajo fue publicado como un resumen de congreso en la LXXV Reunión Anual de la Sociedad Española de Neurología (ID: 19346).

## Contribuciones de los Autores

EMC y RLI diseñaron el estudio de investigación. EMC llevó a cabo la investigación. JSRDG brindó apoyo y asesoramiento sobre los análisis estadísticos. EMC y PTA analizaron los datos. EMC, JSRDG y PTA redactaron el manuscrito. Todos los autores contribuyeron a los cambios editoriales del manuscrito. Todos los autores leyeron y aprobaron el manuscrito final. Todos los autores han participado lo suficiente en el trabajo y han acordado ser responsables de todos los aspectos del mismo.

## Aprobación Ética y Consentimiento Informado

El estudio se llevó a cabo de acuerdo con las directrices de la Declaración de Helsinki y fue aprobado por el Comité Ético de Investigación Clínica de Navarra (PI_2023/71). Debido a que la información utilizada para la creación de la base de datos sobre la que se desarrollará el proyecto se ha obtenida a través de la solicitud de los datos anonimizados de las fichas de “biomarcadores enfermedad de Alzheimer” de la HCI y que los datos a analizar forman parte de la práctica clínica habitual, por lo tanto, se renuncia al consentimiento informado.

## Agradecimientos

No aplicable.

## Financiación

Esta investigación no recibió financiación externa.
